# Binocular misalignments elicited by altered gravity provide evidence for nonlinear central compensation

**DOI:** 10.3389/fnsys.2015.00081

**Published:** 2015-06-02

**Authors:** Kara H. Beaton, W. Cary Huffman, Michael C. Schubert

**Affiliations:** ^1^Department of Otolaryngology – Head and Neck Surgery, The Johns Hopkins University School of MedicineBaltimore, MD, USA; ^2^Department of Mathematics and Statistics, Loyola UniversityChicago, IL, USA; ^3^Department of Physical Medicine and Rehabilitation, The Johns Hopkins University School of MedicineBaltimore, MD, USA

**Keywords:** oculomotor, otolith, parabolic flight, gravity, model

## Abstract

Increased ocular positioning misalignments upon exposure to altered gravity levels (g-levels) have been strongly correlated with space motion sickness (SMS) severity, possibly due to underlying otolith asymmetries uncompensated in novel gravitational environments. We investigated vertical and torsional ocular positioning misalignments elicited by the 0 and 1.8 g g-levels of parabolic flight and used these data to develop a computational model to describe how such misalignments might arise. Ocular misalignments were inferred through two perceptual nulling tasks: Vertical Alignment Nulling (VAN) and Torsional Alignment Nulling (TAN). All test subjects exhibited significant differences in ocular misalignments in the novel g-levels, which we postulate to be the result of healthy individuals with 1 g-tuned central compensatory mechanisms unadapted to the parabolic flight environment. Furthermore, the *magnitude* and *direction* of ocular misalignments in hypo-g and hyper-g, in comparison to 1 g, were *nonlinear* and *nonmonotonic*. Previous linear models of central compensation do not predict this. Here we show that a single model of the form *a* + *bg*^ε^, where *a*, *b*, and ε are the model parameters and *g* is the current g-level, accounts for both the vertical and torsional ocular misalignment data observed inflight. Furthering our understanding of oculomotor control is critical for the development of interventions that promote adaptation in spaceflight (e.g., countermeasures for novel g-level exposure) and terrestrial (e.g., rehabilitation protocols for vestibular pathology) environments.

## Introduction

Spaceflight elicits adaptive changes across all physiological systems (White, 1998). In particular, the altered gravity levels (g-levels) modulate otolith signaling, disrupting multiple sensorimotor subsystems simultaneously until the central nervous system (CNS) becomes properly calibrated to the current g-level (Young et al., 1986; Reschke et al., 1994). Inflight, astronauts experience miscalibrated otolith-ocular reflexes, reduced eye-hand coordination and fine motor control, spatial disorientation, and perceptual illusions (Reschke et al., 1996; Clément and Reschke, 2008). Space motion sickness (SMS) remains one of the most significant and unpredictable operational challenges of spaceflight, affecting over two-thirds of all astronauts (Davis et al., 1988). Upon Earth-return, crewmembers express clumsiness in their movements, persisting sensation aftereffects, standing and walking vertigo, nausea, difficulty concentrating, and blurred vision (Bacal et al., 2003).

Studying sensorimotor responses in altered gravity and developing computational models for how the CNS facilitates adaptation to such environments will allow us to quantify physiologic commonalities and individual differences. With the prospect of longer duration missions beyond low-Earth orbit, such knowledge is vital to maximize crew safety and mission success. Countermeasures must be developed to enable astronauts to live in space for prolonged periods of time during transit and prepare them to re-enter a gravitational field upon arrival at their destination. This means that crewmembers will require appropriate technologies to perform accurate self-assessments and rehabilitations inflight. Therefore, the two objectives of this work were to (1) evaluate a novel, portable device for quantifying ocular positioning misalignments during altered-gravity exposure, and (2) explore through the development of a computational model how oculomotor pathways might be organized such that these misalignments arise.

Increased binocular positioning misalignments upon exposure to novel gravitational environments have been strongly linked to SMS susceptibility. For instance, Kornilova and colleagues reported a strong correlation between SMS and post-flight ocular counterroll (OCR) asymmetry in Russian cosmonauts (Kornilova et al., 1983): eleven out of twelve long-duration crewmembers (missions between 30 and 211 days) who experienced SMS inflight also exhibited asymmetries in OCR post-flight. Vogel and Kass measured OCR elicited by leftward and rightward roll tilts up to 90° in four Spacelab-1 crewmembers pre- and post-mission (Vogel and Kass, 1986); the astronaut most prone to SMS expressed the largest asymmetry in OCR gain preflight, while the astronaut least susceptible showed symmetrical OCR gain preflight and quickly returned to baseline levels post-flight. Diamond and Markham performed a series of experiments on astronauts in which they correlated asymmetries in binocular torsion during the altered g-levels of parabolic flight with SMS experience (Diamond et al., 1990; Diamond and Markham, 1991, 1992b); astronauts who exhibited the largest differences in torsional asymmetry between the 0 and 1.8 g phases of parabolic flight were the ones who endured the most severe SMS during their missions.

The relationship between ocular positioning misalignments and SMS poses an important question: Can measures of binocular alignment enable preflight predictions of inflight motion sickness susceptibility and symptom severity? Such results could facilitate the development of individually tailored training protocols and countermeasures. Hence, the first aim of this study was to validate a novel, hand-held apparatus to quantify ocular positioning misalignments in the altered g-levels of parabolic flight^1^. Unlike the techniques employed by previous investigators, our approach does not incorporate direct measures of eye movements, thereby eliminating delicate, uncomfortable equipment and computationally expensive algorithms, which are non-ideal for spaceflight operations. Instead, we have developed perceptual misalignment-nulling tasks to infer ocular positioning misalignments that can be quickly self-administered using portable equipment (Beaton et al., 2013). Furthermore, our device fully eliminates binocular visual cues, which are known to mask ocular positioning misalignments (Burian, 1939; Ogle and Prangen, 1953; Kertesz and Jones, 1970; Crone and Everhard-Hard, 1975; Kertesz, 1981; Guyton, 1988; Paterson et al., 2009). Previous investigators have captured ocular positions during altered g-level exposure using film cameras in ambient light (Vogel and Kass, 1986; Diamond et al., 1990; Diamond and Markham, 1991, 1992b), and hence the binocular visual cues available to the subjects during testing may have interfered with their ocular misalignment results. We hypothesized that the ocular misalignments quantified through our perceptual nulling tasks would exhibit similar g-level dependencies observed by previous investigators, but may be more accurate and consistent due to the absence of binocular visual cues.

One plausible explanation for why increased ocular misalignments might arise upon exposure to novel g-levels is due to inherent asymmetries between the left and right otolith systems. It is conceivable that nature does not (and cannot) produce precisely identical otoconial maculae on both sides of the head, and so small anatomical asymmetries may exist in at least some people (Yegorov and Samarin, 1970; von Baumgarten and Thümler, 1979). Furthermore, asymmetries in hair cell counts, sensitivities, or distributions, or in the neural relationships between primary afferents and their receptors may also occur (Bracchi et al., 1975; Markham and Diamond, 1993). During early development on 1 g Earth, central processes regulate these asymmetries to mitigate functional vestibular deficits. While it is difficult to measure otoconial mass asymmetries in vertebrates due to surrounding temporal bone and loss of otoconia during specimen preparation (Scherer et al., 2001), such asymmetries have been confirmed in various species of fish. Additionally, numerous studies have demonstrated a correlation between increased otoconial mass asymmetries in fish and pathologic swimming patterns and behavior (e.g., lethargy and emesis) during centrifugation, parabolic flight, and spaceflight (von Baumgarten et al., 1972; Wetzig, 1983; Ijiri, 1995; Scherer et al., 1997; Anken et al., 1998; Hilbig et al., 2002; Helling et al., 2003).

In humans, asymmetries in oculomotor behavior can be examined as evidence of anatomical or physiological otolith asymmetries. Among other roles, the otolith organs control vertical and torsional eye movements as related to gravito-inertial accelerations (GIA), and hence underlying otolith asymmetries may manifest as vertical and torsional binocular positioning misalignments in novel (uncompensated) gravity environments when binocular visual cues are eliminated (Uchino et al., 1996; Isu et al., 2000; Newlands et al., 2003; Goto et al., 2004; Highstein and Holstein, 2006). Measures of binocular torsion have been the primary eye movement of choice (Lackner et al., 1987; Wetzig et al., 1990; Diamond and Markham, 1991, 1992a,b; Cheung et al., 1992; Markham and Diamond, 1992, 1993; Wuyts et al., 2003), possibly because torsion is a reflexive, vestigial eye movement that, unlike vertical eye movements, is not subject to voluntary control (Collewijn et al., 1988; Misslisch et al., 2001). Nonetheless, we examined both vertical and torsional ocular positioning misalignments in this study.

In 1979, von Baumgarten and Thümler proposed a simple model for vestibular adaptation in altered gravity environments based on the hypotheses that (1) otolith asymmetries are present in at least some individuals, and (2) these asymmetries are centrally compensated through additional neural impulses stemming from the brainstem reticular formation and/or cerebellum (von Bechterew, 1909; Yegorov and Samarin, 1970; Schaefer and Meyer, 1974). Under this model, people with larger left-right asymmetries would require greater compensatory adjustments following exposure to novel g-levels. von Baumgarten and Thümler posited that such individuals might therefore experience functional vestibular deficits that are more extreme or that persist longer during the adaptation process. The second aim of this study was to consider our ocular misalignment results in light of the von Baumgarten and Thümler central compensation model. While our data characterize oculomotor behavior, they do not explain how neurophysiological pathways might be organized to produce such results. Understanding the underlying neural circuitry through the development of mathematical models may enable us to pair changes in specific neurophysiological structures with changes in behavior, which has important implications for various interventions, such as rehabilitation prescriptions for terrestrial pathologies or countermeasures for astronauts. The von Baumgarten and Thümler model does not, however, account for all of the ocular misalignment data collected in altered g-levels to-date. But it does provide a simple framework from which we developed a more generalized model that better describes g-level dependent ocular misalignments. We predicted that our model would be robust to support both the vertical and torsional ocular misalignments observed during altered g-level exposure.

## Materials and methods

### Test subjects

Six healthy individuals with no known vestibular, oculomotor, or neurological deficits volunteered as test subjects. All provided written, informed consent to a protocol pre-approved by the Johns Hopkins Medicine and the NASA Johnson Space Center Institutional Review Boards. Five of the subjects were naïve to the objectives of this study. Four of the subjects were naïve to the parabolic-flight environment itself (i.e., had never previously flown in parabolic flight). None of the subjects took any anti-motion sickness medications preflight (including the scopolamine offered by NASA flight surgeons), as these drugs may inhibit sensorimotor function (Pyykko et al., 1985; Davis et al., 1993; Shojaku et al., 1993). To ensure that our test subjects would be able to withstand the provocative nature of the flights and provide sufficient inflight validation data, we preferentially included individuals with a high tolerance for terrestrial motion sickness.

### Quantifying binocular positioning misalignments

Binocular positioning misalignments were quantified through two perceptual nulling tasks: Vertical Alignment Nulling (VAN) and Torsional Alignment Nulling (TAN). In VAN and TAN, subjects hold a tablet computer that displays one horizontal red line and one horizontal blue line while viewing through color-matched red and blue filters; this provides separate visual information to each eye. One of these horizontal lines, designated as the *stationary line*, remains fixed on the screen, while the other, the *moving line*, is repositioned by the subject vertically during VAN or rotationally during TAN. The subject's objective is to adjust the moving line until it appears perfectly in-line with the stationary line (in other words, null any visually apparent vertical or rotational offset between the two lines). If there exists a small range of positions for which the moving line appears aligned with the stationary line, indicating that the subject can perceptually fuse a slight physical offset between the two lines, then the subject is instructed to find the middle of this range. The final amount by which the lines are separated from one another vertically or rotated relative to one another provides a perceptual measure of vertical or torsional ocular misalignment, respectively. For example, if a subject sets the right line above the left line by 2° during VAN, then we infer that this individual has a vertical misalignment such that the right eye is elevated 2° above the left eye (i.e., the right fovea is elevated 2° above the left fovea). If a subject orients the right line 2° clockwise relative to the left line during TAN, then we infer that this individual has a torsional misalignment such that the right eye is extorted 2° relative to the left eye. If a subject perfectly aligns the two lines during both VAN and TAN, then we infer that this individual has perfect vertical and torsional binocular alignment. By convention, a positive vertical misalignment indicates that the right eye is depressed relative to the left eye, and a positive torsional misalignment indicates that the right eye is extorted relative to the left eye. During testing, the tablet-to-subject distance is fixed at 0.42 m by a tether extending from the back of the tablet to the subject.

Importantly, all VAN and TAN testing is performed in complete darkness under a shroud to ensure that extraneous visual cues do not mask the binocular misalignments (Burian, 1939; Ogle and Prangen, 1953; Kertesz and Jones, 1970; Crone and Everhard-Hard, 1975; Kertesz, 1981; Guyton, 1988; Paterson et al., 2009). Active-matrix organic light-emitting diode (AMOLED) tablet technology allows only the designated pixels on the tablet (i.e., only the red and blue lines) to be illuminated so that any binocular visual artifacts, such as the rectangular tablet screen backlight visible on traditional LCD screens, are not present. During testing, subjects interact with the tablet through vibrotactile control buttons, as opposed to visual cues.

### Experimental approach

In parabolic flight, a specially outfitted aircraft flies a parabolic trajectory that provides alternating levels of 0 and 1.8 g, as perceived by the passengers inside. Each 0 and 1.8 g phase lasts approximately 25 and 40 s, respectively, and transitions between cycles are brief (< 1 s). Further details regarding the aircraft dynamics and flight controls have been described by Karmali and Shelhamer (2008). All data for our experiments were collected during 1 week of parabolic flights, in which each subject flew a single flight. Each flight encompassed thirty 0 g parabolas.

Subjects were trained on VAN and TAN several days prior to their respective flights. Training included practicing the VAN and TAN tasks first in the light, so that investigators could verify that test instructions were understood, and then in complete darkness under the shroud. Approximately 50 trials of VAN and TAN were completed during this training session. Training was refreshed the morning of the flight. Baseline 1 g data was collected onboard the aircraft approximately 1 h prior to takeoff and consisted of 20 trials each of VAN and TAN.

Inflight, VAN and TAN data were collected during four five-parabola blocks at the beginning and end of flight: the *Early* TAN test block occurred during parabolas 1–5, the *Early* VAN test block occurred during parabolas 6–10, the *Late* VAN test block occurred during parabolas 21–25, and the *Late* TAN test block occurred during parabolas 26–30. For each test block, subjects were instructed to perform as many successive VAN or TAN trials as possible. Synchronized three-axis accelerometer data was collected simultaneously so that the VAN and TAN trials could be separated by g-level during post-flight analysis. During testing, subjects were loosely strapped to the floor of the aircraft to enable gentle rising and falling with the g-levels. For the parabolas between the *Early* and *Late* test blocks (parabolas 11–20), subjects were removed from the shroud and floor straps and allowed to free-float inside the cabin, thereby enabling full immersion, and possibly adaptation, to the parabolic flight environment. During the aircraft's 1 g turns and any straight-and-level breaks, subjects rested outside of the shroud. If at any point subjects began to experience motion sickness symptoms, including hot or cold sweats, stomach awareness, dizziness, or nausea, testing was stopped immediately and subjects were removed from the shroud and instructed to close their eyes and rest.

### Modeling approach

In this paper, we develop a model that describes the increased ocular misalignments observed in our subjects upon initial exposure to novel g-levels. Previous models, including the one proposed by von Baumgarten and Thümler, do not adequately capture our inflight results; hence a new model is needed. Our underlying hypothesis is that our observed misalignments are the result of innate otolith asymmetries that are centrally compensated in 1 g, but uncompensated in non-1 g until appropriate neural adjustments are learned for that g-level through adaptation. Our model is based on the one proposed by von Baumgarten and Thümler, which we now describe in brief to define the terms and equations that will be extended to our model in Sections Incorporating a Nonlinear Gravity Component into Central Compensation Facilitates the Parabolic Flight Results and Specifying the Central Compensation Inputs. Differences between the von Baumgarten and Thümler model and our model are detailed in Section Incorporating a Nonlinear Gravity Component into Central Compensation Facilitates the Parabolic Flight Results.

**Figure 1:**
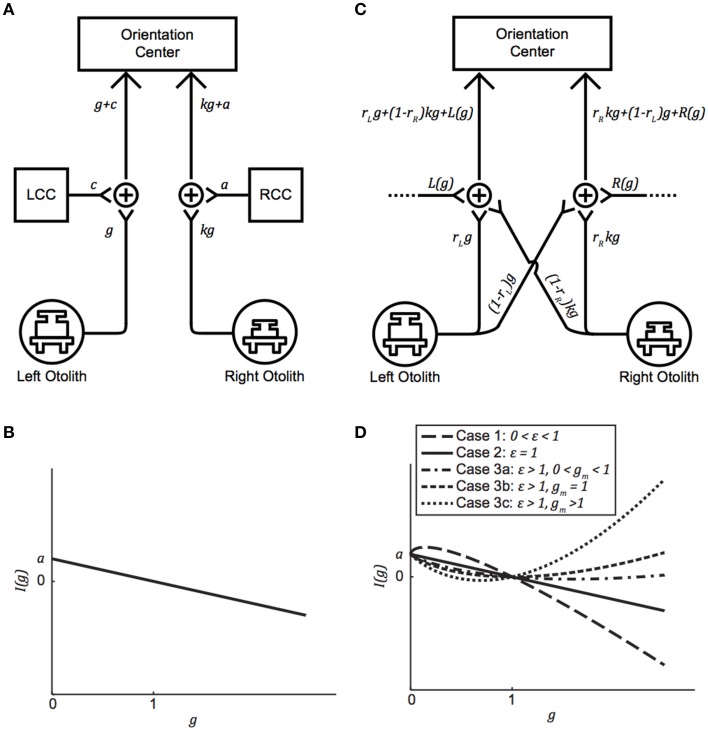
**(A)** Generalized version of the von Baumgarten and Thümler model for central compensation of an otolith asymmetry. *g* is the current g-level, *k* is the otolith asymmetry parameter (0 < *k* < 1), LCC, and RCC are the left and right compensation centers, respectively, and *a* and *c* are the amounts of additive neural compensation required for balance between the left and right sides (*a*, *c* ≥ 0). **(B)** Imbalance as a function of g-level for the von Baumgarten and Thümler model. The direction of imbalance experienced upon an initial change in g-level depends on whether the new g-level is hypo-g or hyper-g. **(C)** Proposed model for compensation of an inherent otolith asymmetry. *r_L_* and *r_R_* are the preponderance parameters (0 < *r_L_*, *r_R_* < 1) and *L*(*g*) and *R*(*g*) facilitate compensation in novel g-levels. **(D)** Possible curves for imbalance as a function of g-level, based on the relationships among model parameters, for the proposed model when *R*(*g*) = *a* + *b*(*g*)^ε^. Note that **(A)** and **(C)** are simply model block diagrams and are not meant to represent neuro-anatomical pathways.

In their model, von Baumgarten and Thümler posit two compensating centers, one on the left and one on the right, and an orientation center that compares the left and right afferent information to generate an overall central vestibular percept (von Baumgarten and Thümler, 1979; Diamond and Markham, 1998; Clarke et al., 1999; Kondrachuk, 2003). This is depicted in Figure 1A, where *g* is the current g-level, *k* is the otolith asymmetry parameter describing the anatomical or physiological asymmetry between the left and right otolith systems (0 < *k* < 1), LCC and RCC represent the left and right compensation centers, respectively, and *a* and *c* are the amounts of additive^2^ neural compensation required for a balanced perception of orientation (*a*, *c* ≥ 0). By convention, compensation is defined to be positive and stemming solely from one side; a negative compensation is interpreted as a positive compensation of the same magnitude from the other side. Under this model, individuals with larger left-right asymmetries require greater compensatory adjustments to *a* and *c* to facilitate a balanced orientation center in novel g-levels.

The magnitude and direction of otolith asymmetry is derived from comparisons of the signals emanating from the left and right sides. In the balanced 1 g condition, *a* > 0, *c* = 0, and the otolith signal from the left is equivalent to the otolith signal from the right:

g+0=kg+a⇒1=k+a⇔k=1−a.

(In the event that the asymmetry parameter *k* is instead on the left side, then *a* = 0, *c* > 0, and *k* = 1 − *c*.) We define an *imbalance function I*(*g*) to quantify the difference in neural signaling between the left and right sides upon an *immediate* change in g-level (i.e., prior to any adaptation):

(1) I(g)=(kg+a)−(g+0)=a(1−g).

If *I*(*g*) = 0, then the left and right sides are balanced (e.g., when *g* = 1 at baseline). If *I*(*g*) > 0, then the signal on the right is stronger than the signal on the left, and if *I*(*g*) < 0, then the signal on the left is stronger than the signal on the right. We denote *I*(*g*) > 0 by the symbol ↶, and *I*(*g*) < 0 by the symbol ↷. Graphing Equation (1) reveals how the direction of imbalance is dictated by g-level: If an individual is suddenly placed in a novel hypo-g environment, then the direction of imbalance is positive (i.e., ↶), whereas if this individual is instead suddenly placed in a novel hyper-g environment, the direction of imbalance is negative (i.e., ↷) (Figure 1B). Under the von Baumgarten and Thümler model, *I*(*g*) is *linear* and *monotonic* for *g* ≥ 0.

The von Baumgarten and Thümler model also describes how much additive neural compensation is needed to facilitate adaptation to a novel g-level, and to which side this compensation must be added. Immediately upon a change from *g* = 1 to *g* = *G*, *I*(*G*) = *a*(1 − *G*). Adaptation is achieved when *c* = 0 is modified to a new value:

$$\begin{array}{l} \left(kG+a\right)-\left(G+c\right)\;=\;\left(1-a\right)G+a-G-c=0\\ \;\Rightarrow c=a\left(1-G\right).\; \end{array}$$

If the novel g-level is *G* = 0 g, for example, then adaptation is achieved when *c* = *a*. If the novel g-level is instead *G* = 2 g, then adaptation is achieved when *c* = − *a*. this makes the LCC compensation negative, which we interpret as a positive compensation by the RCC of amount *a*.

## Results

### Binocular misalignments increase in novel g-levels

**Figure 2:**
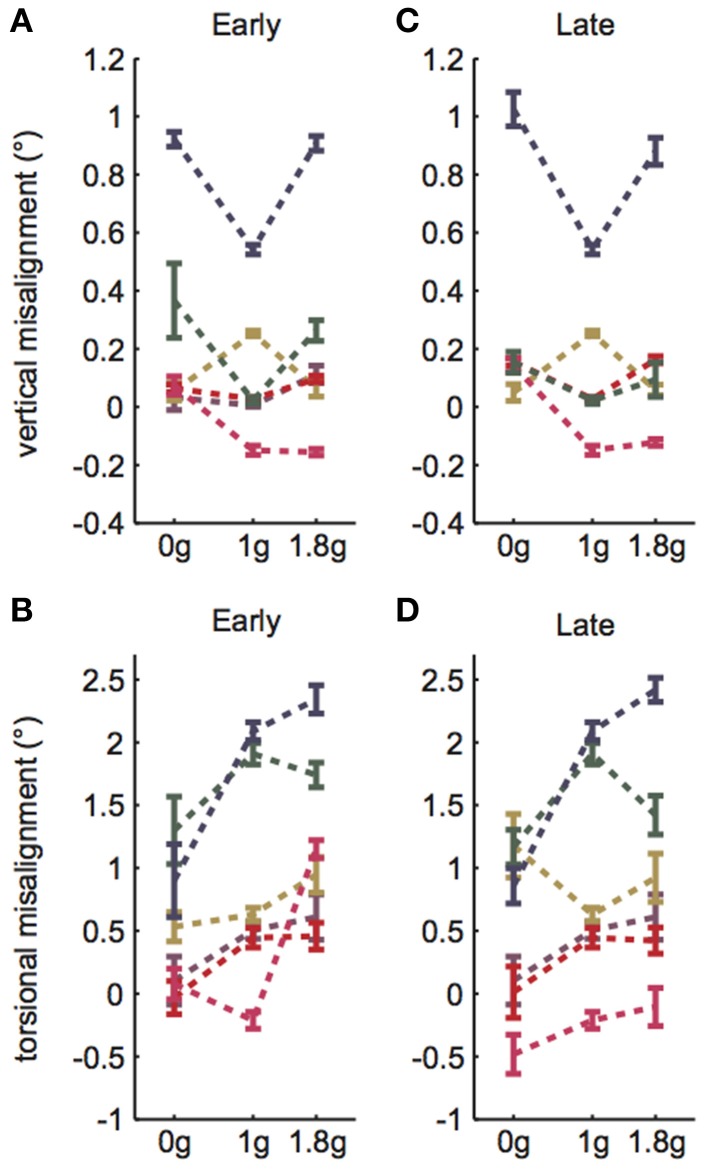
**VAN and TAN parabolic flight results. (A)** *Early* VAN data was collected during parabolas 6–10. **(B)** *Early* TAN data was collected during parabolas 1–5. **(C)** *Late* VAN data was collected during parabolas 26–30. One subject was not tested during these parabolas. **(D)** *Late* TAN data was collected during parabolas 21–25. Dashed lines simply group data within subjects and do not represent actual (or proposed) g-level responses. Error bars are ±1SE.

Subjects performed VAN and TAN during the *Early* and *Late* test blocks, completing approximately 15–25 trials during the five hypo-g phases and 25–40 trials during the five hyper-g phases of each test block. The mean VAN and TAN results collected *Early* and *Late* are displayed in Figure 2; note that the dashed lines simply group data within subjects and do not represent actual (or proposed) g-level responses. Five of the six test subjects completed both the *Early* and *Late* tests without experiencing any motion sickness symptoms. One subject elected to forgo his *Late* VAN test due to feeling excessively warm under the shroud; this individual did not experience any other symptoms, nor did his symptoms worsen while he rested outside of the shroud.

**Figure 3:**
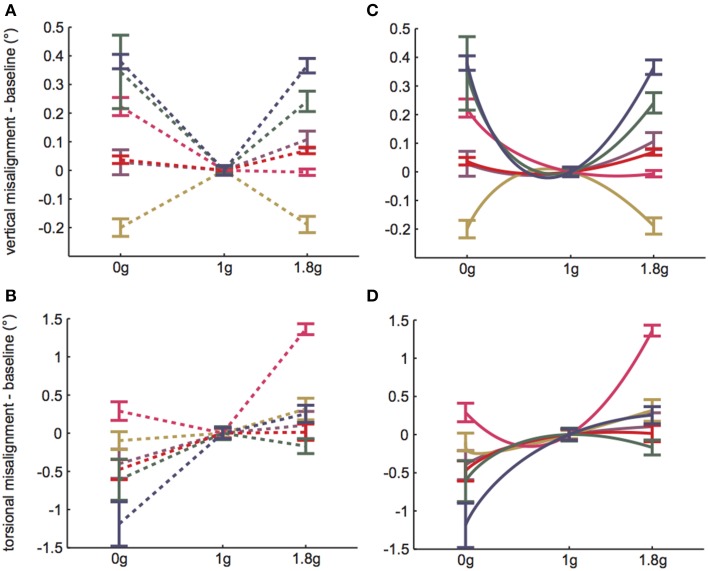
**1 g baseline data subtracted from *Early* parabolic flight data for (A) VAN and (B) TAN**. Dashed lines simply group data within subjects and do not represent actual (or proposed) g-level responses. **(C,D)** Solid lines represent model predictions, given *b* = 4*a*. Error bars are ±1SE.

A linear random intercept model was fit to estimate the mean ocular misalignments observed at baseline (1 g) and at each combination of time (*Early* and *Late*) and non-1 g gravity level (0 and 1.8 g). The random intercept was included to account for the correlation in observed ocular misalignments within a subject under the various conditions. As expected, the model revealed significant g-level dependent differences in 1 g vs. non-1 g responses (VAN: χ^2^(1) = 36.7, *p* << 0.01; TAN: χ^2^(1) = 84.4, *p* << 0.01). Our primary metric of interest was whether subjects expressed a *change* in ocular misalignments in novel gravity conditions, as this could be representative of an underlying otolith asymmetry masked by central compensation in 1 g. Note that a decrease in ocular misalignment (smaller numerical values on the graphs) in non-1 g compared to 1 g should be interpreted as a change in the directional orientation of one eye relative to the other in this new g-level. For example, recall that we infer a positive TAN value to mean that the right eye is extorted with respect to the left eye in 1 g. Hence, a smaller positive TAN value in a novel g-level means that the right eye is now more intorted than it is in 1 g. Since none of our subjects express vestibular performance decrements in everyday life, we presume that any ocular misalignments present in their baseline 1 g tests are precisely what their individual vestibular systems deem “balanced” or “nominal;” furthermore, because these baseline misalignments are too small to elicit functional visual deficits, there is little incentive for the CNS to adjust these responses (e.g., expend more neural “effort” to generate misalignments closer to 0.0 in 1 g). We graph these non-1 g to 1 g differences directly in Figures 3A,B.

The linear random intercept model also revealed moderately significant differences *Early* vs. *Late* (VAN: χ^2^(1) = 6.4, *p* = 0.04; TAN: χ^2^(1) = 7.7, *p* < 0.01). However, these differences were on the order of hundredths of a degree for VAN and tenths of a degree for TAN; such differences are well within the physiological range for repeated-measures testing of similar behavioral responses (Tarnutzer et al., 2009, 2012) and are also an order of magnitude less than the more important differences observed between 1 g and non-1 g responses. Hence, the VAN and TAN results were relatively comparable during *Early* vs. *Late* test sessions, despite subjects experiencing a ten-parabola adaptation period in between. This means that subjects were consistent in their reporting from the start of their first 0 g parabola to the end of their thirtieth 0 g parabola (in terms of both average responses and variability among the individual trials) and that relatively little adaptation occurred over the course of the flight. If adaptation had occurred, we would expect the ocular misalignments to trend toward the preflight 1 g levels in the hypo-g and hyper-g phases of flight. However, this was not (statistically) observed for our subjects.

For the remainder of the paper we focus on the VAN and TAN results obtained during the *Early* test sessions, as the *Early* condition represents subjects' initial exposure to 0 and 1.8 g. Figures 3A,B highlight the statistically significant changes in ocular misalignments in non-1 g environments, which agree with the von Baumgarten and Thümler prediction of 1 g-tuned central compensatory mechanisms that are inappropriate for non-1 g environments prior to adaptation. Our data do, however, differ from the von Baumgarten and Thümler predictions of linear, monotonic changes in ocular misalignments with increasing *g* for *g* ≥ 0 (Figure 1B); while the TAN results from some subjects do follow the monotonic trend, the TAN results from other subjects and the VAN results from all subjects do not. Therefore, a new model, adapted from the von Baumgarten and Thümler version, has been developed to account for these new data.

### Incorporating a nonlinear gravity component into central compensation facilitates the parabolic flight results

**Figure 4:**
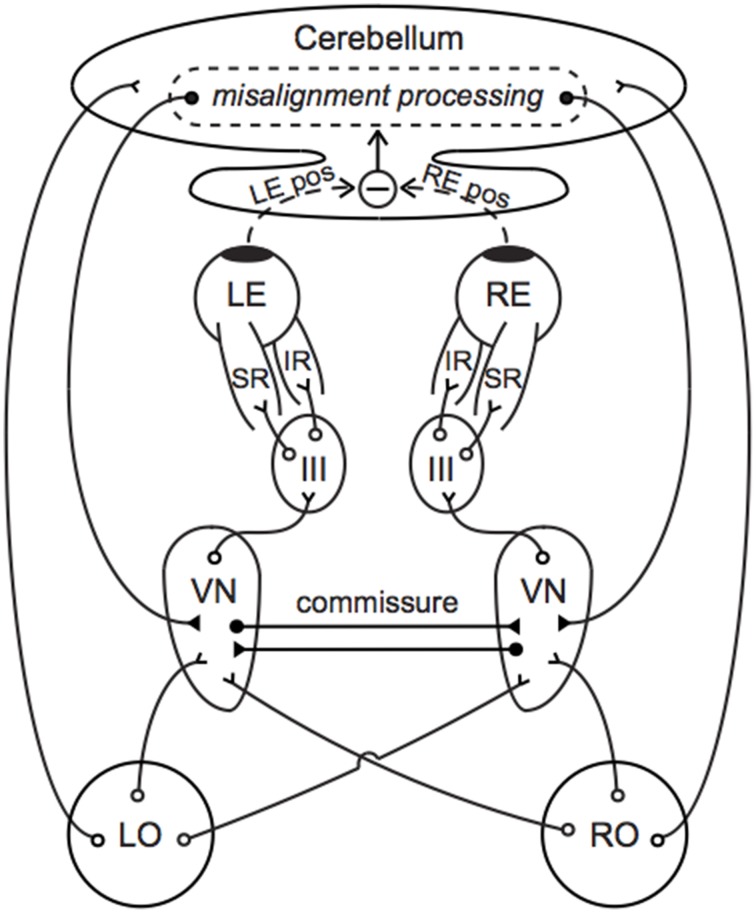
**Neural compensation for an otolith asymmetry manifest as a vertical ocular positioning misalignment**. Primary afferents synapse in the VN, which project to III to control contraction of the SR and IR. The cerebellum compares the left and right eye positions and sends compensatory signals back to the VN. Commissural connections between the left and right VN improve the sensitivity of otolith-mediated reflexes and may further aid in compensation. Direct projections between the end organs and cerebellum facilitate an immediate transmission of the current GIA. Open circles indicate excitatory pathways and filled circles indicate inhibitory pathways. LO, left otolith; RO, right otolith; VN, vestibular nucleus; III, oculomotor nucleus; LE, left eye; RE, right eye; LE pos, left eye position; RE pos, right eye position.

Our new model is presented in Figure 1C; note that although certain anatomical structures are implicit in this block-diagram representation, a more detailed neurophysiological description is provided in Figure 4 and Section Gravity-dependent Ocular Misalignments Addressed through Nonlinear Central Compensation. The otolith asymmetry parameter *k* (0 <*k* < 1) describes the anatomical or physiological asymmetry between the left and right otolith systems^3^. Our model also incorporates preponderance parameters *r_L_* and *r_R_* (0 ≤ *r_L_*, *r_R_* ≤ 1) to describe the relative ratio of ipsilateral-to-contralateral innervation between the end organs and central targets; values of 1.0 are representative of end organs that send 100% of their projections to the ipsilateral side, while values of 0.0 indicate 100% projections to the contralateral side^4^. Fernandez and Goldberg (1976a) measured *r_L_* and *r_R_* to be 0.75 in the squirrel monkey utricle; to the best of our knowledge, *r_L_* and *r_R_* have not yet been quantified for the saccule, but they are presumed here to exist.

Instead of RCC and LCC, which connote separate mechanisms for left vs. right compensation, our model specifies left and right gravity-dependent central compensation inputs *L*(*g*) and *R*(*g*) to better align with current models of central compensation (Büttner-Ennever and Gerrits, 2004; Faulstich et al., 2006); sources for these inputs are discussed in Section Gravity-dependent Ocular Misalignments Addressed through Nonlinear Central Compensation. Upon exposure to a novel g-level, parameters within *L*(*g*) and *R*(*g*) are modified so that adaptation to the new gravitational environment can be achieved. While the von Baumgarten and Thümler model treats these functions as constants, gravity-dependent variants are consistent with the vertical and torsional misalignment data we observed inflight. Analogous to Figure 1A, we depict *k* on the right in Figure 1C, and hence *R*(*g*) > 0 and *L*(*g*) = 0; should *k* instead be on the left, all that is needed is to reverse the diagram and the following computations remain the same.

The amount of imbalance between the left and right sides in Figure 1C is

(2) $$\;I\left(g\right)=\left[r_{R}kg+\left(1-r_{L}\right)g+R\left(g\right)\right]-\;\left[r_{L}g+\left(1-r_{R}\right)kg\right].$$

As described in Section Modeling Approach, we assume that *I*(1) = 0 for healthy individuals and denote *I*(*g*)> 0 by ↶ and *I*(*g*)< 0 by ↷.

The particular form of *R*(*g*) will dictate the amount and direction of imbalance observed in different g-levels. There are a variety of *R*(*g*) functions that reproduce our ocular misalignment data, and one reasonable form is discussed in the following section. Because *I*(1) = 0, the only restriction on *R*(*g*) is that it satisfy

(3) $$R\left(1\right)=2r_{L}-2r_{R}k+k-1,$$

which is obtained by setting *g* = 1 in (2).

Simplifying (2) and using (3), we obtain

(4) I(g)=R(g)−gR(1).

This shows that our model has the following important property: both the amount and direction of imbalance are independent of the preponderance parameters *r_L_* and *r_R_* once *R*(*g*) is known and satisfies (3).

Our model also supports the well-known “re-adaptation” phenomenon, in which re-adaptation to 1 g (following adaptation to some non-1 g environment) generates imbalance in the opposite direction (Young et al., 1984; Parker et al., 1985; Correia, 1998). von Baumgartner and Thümler presume that adaptation is achieved by the contralateral side supplying additional neural impulses to the orientation center until balance in the new gravity environment is attained (von Baumgarten and Thümler, 1979). Suppose a healthy individual is exposed to a novel g-level *g* = *G* ≠ 1 and that this g-level generates ↶ imbalance; hence, *I*(*G*) > 0. In our model, analogous to the von Baumgarten and Thümler model, adaptation drives a modification to the left input from *L*(*g*) = 0 to a new left input L^*^(*g*) > 0. As such, the new amount of imbalance between the left and right sides is

$$\begin{array}{l} I^{*}\left(g\right)=\left[r_{R}kg+\left(1-r_{L}\right)g+R\left(g\right)\right]\\ \;-\left[r_{L}g+\left(1-r_{R}\right)kg+L^{*}\left(g\right)\right]=I\left(g\right)-L^{*}\left(g\right). \end{array}$$

When adaptation is complete and balance has been restored at *g* = *G* ≠ 1, then I^*^(*G*) = 0. If this individual now returns to 1 g, then

$$I^{*}\left(1\right)=I\left(1\right)-L^{*}\left(1\right)=0-\;L^{*}\left(1\right)=-\;L^{*}\left(1\right)\;<0.$$

So, returning to 1 g following adaptation to some novel g-level *g* = *G* ≠ 1 generates imbalance in the direction opposite to that which was experienced at g-level *g* = *G* ≠ 1.

We know from the parabolic flight and spaceflight literature that time is required to adapt to novel gravity environments (Davis et al., 1988; Baroni et al., 2001; Williams, 2003). In terms of our model, adaptation occurs through modulation of *R*(*g*) and *L*(*g*). In other words, *R*(*g*) and *L*(*g*) are really *R*(*g*,τ) and *L*(*g*,τ), respectively, where τ represents the time constant(s) of adaptation. Adaptation is achieved when *R*(*g*,τ) and *L*(*g*,τ) have been sufficiently modified so that *I*(*g*,τ) = 0. Because our data does not indicate that significant adaptation occurred over the course of a single parabolic flight, we do not yet have enough data to incorporate a time parameter into our model.

### Specifying the central compensation inputs

One reasonable set of central compensation inputs, both mathematically and neurophysiologically, is

$$R\left(g\right)=a+bg^{\varepsilon }$$

and

$$L\left(g\right)=c+dg^{\mu },$$

where *a*, *b*, *c*, *d*, ε, and μ are non-negative free parameters that represent the factors modulated by the CNS to allow for adaptation to novel g-levels. Based on our data, we propose that these parameters are unique to each individual and unique to vertical vs. torsional compensation within a given individual. Assuming only *R*(*g*) provides compensation in 1 g,

$$a+bg^{\varepsilon }>0$$

and

c=d=0 (any μ≥0).

From Equation (4),

(5) $$I\left(g\right)=a+bg^{\varepsilon }-g\left(a+b\right).$$

It should be noted that small changes in *a*, *b*, and ε induce small changes in *I*(*g*). Specifically, using partial derivatives applied to (5), if *a* is changed by Δ*a*, then *I*(*g*) is changed by (1 − *g*)Δ*a*. If *b* is changed by Δ*b*, then *I*(*g*) is changed by (*g*^ε^ −*g*)Δ*b*. If ε is changed by Δε, then *I*(*g*) is changed by *b*ln(*g*)*g*^ε^ Δε. This indicates that the model is robust.

Our specific choice of *R*(*g*) and *L*(*g*) yield the imbalance function *I*(*g*) given in (5). We are interested in two questions regarding *I*(*g*). First, how does our *I*(*g*) in (5) relate to the von Baumgarten and Thümler *I*(*g*) in (1)? Second, for what values of *g* does *I*(*g*) produce ↶ imbalance, ↷ imbalance, or balance? Both are answered by analyzing the shape of the graph of *I*(*g*). This shape depends on the relationships among *a*, *b*, and ε. In our model, *I*(0) must exist, which implies that ε ≥ 0. When ε = 0, *R*(*g*) = *a* + *b*, which is mathematically equivalent to the von Baumgarten and Thümler model because *R*(*g*) is a constant. To describe the remaining shapes of *I*(*g*), we now assume ε> 0, and the derivatives of *I*(*g*) are helpful:

$$\frac{dI}{dg}=-a-b+b\varepsilon g^{\varepsilon -1}$$

and

$$\frac{d^{2}I}{dg^{2}}=b\varepsilon \left(\varepsilon -1\right)g^{\varepsilon -2}.$$

Note that $\frac{dI}{dg}=0$ when $g^{\varepsilon -1}=\frac{a+b}{b\varepsilon }\Rightarrow g^{1-\varepsilon }=\frac{b\varepsilon }{a+b}$, implying that *I*(*g*) has at most one local extremum. We consider three possibilities for ε:

Case 1: 0 < ε < 1

In this case, $\frac{d^{2}I}{dg^{2}}<0$, implying that *I*(*g*) is concave down and has its maximum at $0<g={\left(\frac{b\varepsilon }{a+b}\right)}^{\frac{1}{1-\varepsilon }}<1$ because *b*ε <*a*+*b*. This means that ↶ imbalance occurs when 0 < *g* < 1 and that ↷ imbalance occurs when *g* > 1.

Case 2: ε = 1

In this case, *I*(*g*) = *a*(1−*g*), which is the von Baumgarten and Thümler model [Figure 1B and Equation (1)]. This means that ↶ imbalance occurs when *0 <g< 1* and that ↷ imbalance occurs when *g* > 1.

Case 3: ε > 1

In this case, $\frac{d^{2}I}{dg^{2}}>0$, implying that *I*(*g*) is concave up and its minimum occurs at $0<g=g_{m}={\left(\frac{a+b}{b\varepsilon }\right)}^{\frac{1}{\varepsilon -1}}$. The location of *g_m_* determines the *I*(*g*) curve, and there are three possibilities.

- 0 <*g_m_* < 1
  This occurs precisely when $a+b<b\varepsilon \Leftrightarrow 1+\frac{a}{b}<\;\varepsilon$. Therefore, *I*(*g*) = 0 for two g-values: *g* = 1 and some *g*_0_ < *g_m_*. This means that ↶ imbalance occurs when 0 < *g* < *g*_0_ and *g* > 1 and that ↷ imbalance occurs when *g*_0_ < *g* < 1.
- *g_m_* = 1
  This occurs when $a+b=b\varepsilon \Leftrightarrow 1+\frac{a}{b}=\;\varepsilon$. In this case, I(*g*) ≥ 0 and the only minimum is at *g* = 1. This means that ↶ imbalance occurs for all *g*≠1.
- *g_m_* > 1
  This occurs exactly when $a+b>b\varepsilon \Leftrightarrow 1+\frac{a}{b}>\varepsilon$. Therefore, *I*(*g*) = 0 for two g-values: *g* = 1 and some *g*_0_ > *g_m_*. This means that ↶ imbalance occurs when 0 < *g* < 1 and *g* > *g*_0_ and that ↷ imbalance occurs when 1 < *g* < *g*_0_.

The five possibilities in Cases 1, 2, and 3 are illustrated in Figure 1D. Note that as ε → 1, the graphs for Cases 1 and 3c approach the graph for Case 2 (the von Baumgarten and Thümler model). Similarly, as $\varepsilon \to 1+\frac{a}{b}$, the graphs for Cases 3a and 3c approach Case 3b, the case where ↶ imbalance occurs for all *g*≠1.

In summary, what we learn from our analysis of *I*(*g*) is that the direction of imbalance for a given g-level is dictated precisely by the relationships among the *a*, *b*, and ε model parameters. Furthermore, if we know the values of *a*, *b*, and ε, we can derive exactly which g-levels should generate each of the two directions of imbalance (↶ vs. ↷).

## Discussion

### VAN and TAN provide simple, rapid measures of g-level dependent binocular positioning misalignments

The development of a hand-held apparatus for evaluating ocular misalignments provides a portable technology to assess oculomotor control in a variety of environments. Our parabolic flight results demonstrate one important value of such a device: the apparent independent control of the two eyes cannot be detected using the simpler and more common monocular tests (Markham et al., 2000). The rapid assessment, minimal hardware, and self-administration capabilities make VAN and TAN ideal for evaluating ocular misalignments in the dynamic parabolic flight environment. We presume that these tests would be equally viable in other operational settings, such as remote field testing, bedside clinical testing, or testing onboard the international space station, where time, equipment, and personnel are limited.

Ocular misalignments due to underlying otolith asymmetries are not easily observed in a 1 g environment, as they are likely masked by central compensation. Therefore, one should not expect to necessarily measure larger ocular misalignments during baseline 1 g tests in individuals who might be presumed to have larger otolith asymmetries. Since otolith signaling drives both vertical and torsional eye movements, whose pathways innervate different anatomical structures, we evaluated both vertical and torsional ocular misalignments in parabolic flight. We found that all subjects exhibited vertical and torsional g-level dependent misalignments. Importantly, these results were repeatable early and late inflight, despite a ten-parabola break in between. Hence, we believe that our data represent some underlying neurophysiological mechanism modulated by gravity. Since our test subjects were non-astronauts and did not experience motion sickness symptoms inflight, we could not correlate their ocular misalignments with either terrestrial (including parabolic flight) or SMS susceptibility, as has been done by previous investigators.

One striking feature of our TAN parabolic flight results was that a relative intorsion of the right eye in 0 g and extorsion in 1.8 g was observed for all subjects. We investigated this further by looking for similar trends from previous investigators, but did not find anything conclusive. For example, Vogel and Kass' four crewmembers all showed higher OCR gains during leftward tilts preflight and during rightward tilts post-flight (Vogel and Kass, 1986). Diamond and colleagues, however, found the opposite result in seven non-astronaut subjects: larger OCR was observed during rightward tilts than leftward tilts, and the depressed eye torted more than the elevated eye (Diamond et al., 1979). Lackner and colleagues claimed that their parabolic flight subjects (non-astronauts) who did not experience inflight motion sickness generated larger amounts of OCR during rightward body tilts than leftward ones, and interpreted this as a greater “efficiency” of the left otoliths in generating OCR in individuals who are less prone to motion sickness during exposure to altered g-levels (Lackner et al., 1987). It is interesting that otolith-ocular responses across these different studies were mixed; there were, however, patterns reported within each study. Similarly, we believe that our finding that subjects tended to generate torsional misalignments in the same direction also reflects a within-study pattern, and may be a clue suggesting otolith-ocular responses vary depending on the exposed stimuli.

Previous parabolic flight experiments have indicated that some individuals rapidly adapt to the parabolic flight environment and that these adaptations can be observed within a single flight (Lackner and Graybiel, 1982; Shelhamer et al., 2002). Our VAN and TAN results, however, do not indicate that adaptation of ocular misalignments occurred over the course of a single flight. This may be expected since subjects were minimally exposed to error signals that normally drive adaptation. For instance, the nature of the VAN and TAN tests is such that no binocular error signal is ever presented to the brain: the lines are viewed monocularly, and subjects are tasked with eliminating any perceived visual misalignments. Because VAN and TAN do not involve head movements or visual stimuli to drive adaptation (Nooij et al., 2011; Wood et al., 2011), true adaptation of VAN and TAN responses will only occur when the CNS realizes, through other processes, that otolith-mediated reflexes are miscalibrated. This will then modify how subjects must adjust the relative positioning of the red and blue lines to perceive a single continuous line, but this process will be transparent to the subjects: they will still perceive their completed trials as single, continuous lines, just as they did in the unadapted state. Furthermore, subjects remained stationary under their shrouds and were not exposed to strong visual cues alerting them to their unique surrounding environment (e.g., people floating by upside down); even during the dedicated ten-parabola adaptation period, subjects remained relatively still to minimize their chances of experiencing motion sickness.

Along these same lines, one might expect subjects with prior parabolic flight experience to show smaller ocular misalignments than naïve fliers because previous exposure to the novel g-levels might be recalled through context-specific adaptation or through rapid re-adaptation due to motor-learning savings. However, this was not observed in our two experienced test subjects. This may have been because several years had passed since their most recent previous flight. Hence, even if gravity-dependent context cues had been learned during previous flights, sufficient savings might not have been retained to warrant faster re-adaptation than what was experienced by our four naïve subjects. Our two experienced fliers also happened to be the oldest participants by several decades, and so it is possible that age-related decrements in vestibular adaptive capabilities led to adaptive responses that more closely resemble those of the younger naïve subjects (Paige, 1992).

### Gravity-dependent ocular misalignments addressed through nonlinear central compensation

The motivation to develop our model was to enhance our understanding of ocular positioning misalignments driven by changes in static otolith signaling. As presumed by previous investigators, we believe that the increased ocular misalignments observed in 0 and 1.8 g stem from innate asymmetries between the left and right otolith systems, uncompensated in the parabolic flight environment. However, it is possible that additional or alternative mechanisms elicited our results. For example, the magnitude and direction of misalignments observed in the different g-levels could have instead arisen from gravity acting on eyeballs of slightly different masses or on oculomotor muscles with slightly different pulling strengths. Future experiments and additional models will need to be developed to more definitively identify the physiological driving force behind g-level dependent ocular misalignments. However, our new model is independent of the precise nature of this force: it simply describes the relationship between a gravity stimulus input and ocular misalignment output.

The von Baumgarten and Thümler model predicts a linear, monotonic change in ocular misalignments for *g* ≥ 0 (Figure 1B). As this does not describe our inflight VAN and TAN results, here we propose a simple nonlinear model of the form *a* + *bg*^ε^ that allows for compensation in novel g-levels. We presume that the numerical values of the model parameters vary among individuals. It is also likely that the model parameters vary for vertical vs. torsional control within a given individual, in accordance with the different neural pathways mediating these responses. We can infer some potential numerical values for the model parameters by which our data might have arisen. Note that because we only have two non-1 g data points for each subject and each test direction (vertical vs. torsional), we cannot precisely determine *a*, *b*, and ε for each subject or test. However, if we assume a fixed relationship between two of the three model parameters, we can uniquely determine all three parameters for a given test. For uniformity and illustrative purposes only, we chose the relationship *b* = 4*a*. (Note that many similar relationships that give comparable results exist.) Then,

$$I\left(g\right)=a+4ag^{\varepsilon }-g\left(a+4a\right)=a(1+4g^{\varepsilon }-5g).$$

Solving for ε yields

$$\varepsilon =\frac{\ln\left[\frac{1}{4}\left(\frac{I(g)}{a}+5g-1\right)\right]}{\ln\left[g\right]}.$$

Thus, ε is determined from our numerical values of *a* = *I*(0) and *I*(1.8). Given *b* = 4*a*, the numerical values of *a*, *b*, and ε for each subject's VAN and TAN data are displayed in Table 1. Our model defines compensation as positive and stemming from the right side (recall *R*(*g*)> 0 and *L*(*g*) = 0). Subjects whose *a* values are negative simply mean their compensations stem from the left side; switching the compensation from one side to the other is represented mathematically by reflecting *I*(*g*) over the g-axis. If we graph *I*(*g*) using the data from Table 1, we obtain Figures 3C,D. Had we had more than two non-1 g data points for each VAN and TAN test, numerical estimates of *a*, *b*, and ε would have been determined by a least squares fit to the data.

**Table 1:** Sample numerical values of model free parameters given *b* = 4*a*.

**Test**	**Subject**	***a* = *I*(0)**	***b* = *4a***	***I*(1.8)**	**ε**
VAN	1	0.028	0.113	0.108	1.841
	2	−0.200	−0.799	−0.189	1.369
	3	0.037	0.148	0.070	1.538
	4	0.223	0.891	−0.006	1.173
	5	0.344	1.375	0.241	1.322
	6	0.380	1.520	0.366	1.372
TAN	1	−0.400	−1.602	0.105	1.123
	2	−0.097	−0.388	0.316	0.290
	3	−0.476	−1.906	0.013	1.173
	4	0.289	1.157	1.362	1.966
	5	−0.610	−2.438	−0.169	1.237
	6	−1.189	−4.756	0.253	1.133

Figure 4 provides a simple anatomical illustration of neural compensation for an otolith asymmetry that manifests as a vertical ocular positioning misalignment in a novel g-level. Primary vestibular afferents synapse in the vestibular nuclei (VN), which project to the oculomotor nuclei (III) to control contraction of the superior and inferior rectus muscles (SR and IR). A similar pathway through III, trochlear nuclei (IV), and the superior and inferior obliques describes torsional oculomotor control. Segregation of the vertical and torsional pathways may be present as early as the level of the end organ; there is evidence that ocular torsion is primarily utricular-driven and that vertical positioning is primarily saccular-driven (Suzuki et al., 1969; Fluur, 1970; Fluur and Mellstrom, 1970a,b; Uchino et al., 1996; Isu et al., 2000; Goto et al., 2004).

Otolith-ocular pathways are enhanced through cerebellar circuitry and commissural connections. Direct projections from the otoliths to the ipsilateral cerebellar nodulus and uvula are well established (Precht and Llinas, 1969; Korte and Mugnaini, 1979; Carleton and Carpenter, 1984; Kevetter and Perachio, 1986; Barmack et al., 1993; Purcell and Perachio, 2001); as many as 70% of primary vestibular afferents are estimated to synapse in the cerebellum (Goldberg et al., 2012b). This feature might enable the current g-level to be a direct parameter in our model's central compensation input functions *L*(*g*) and *R*(*g*). The cerebellum determines ocular misalignment through visual disparity cues and proprioceptive feedback from the eye muscles (Fuchs and Kornhuber, 1969; Baker et al., 1972; Donaldson and Hawthorne, 1979; Zee et al., 1981), in conjunction with information from primary and secondary vestibular afferents. This misalignment information is fed back to the VN through direct, bilateral inhibitory projections of cerebellar Purkinje cells (Batton et al., 1977; Noda et al., 1990). Inhibitory commissural connections between the ipsilateral and contralateral VN amplify asymmetries between the left and right sides, which has been suggested to improve the sensitivity and resolution of otolith-mediated processes (Uchino et al., 1999; Karmali, 2007).

Our *R*(*g*) parameters *a*, *b*, and ε represent additive, multiplicative (gain control), and exponential transformations, respectively, and are routinely observed in single neurons and within larger neural networks in the brainstem and cerebellum (Fernandez and Goldberg, 1976a; Chadderton et al., 2004; Silver, 2010; Hildebrandt et al., 2011). These parameters might be computed and adapted in the flocculus and paraflocculus, which have been linked to the generation and plasticity of compensatory eye movements (Marr, 1969; Albus, 1971; Faulstich et al., 2006; Goldberg et al., 2012a), and further refined in the VN through interneurons and crossed commissural connections (Miles and Lisberger, 1981; Büttner-Ennever and Gerrits, 2004). Furthermore, it is possible that the nonlinear amplifications of the GIA are performed by the primary afferents themselves, especially for g-levels near zero and substantially greater than one, as evidenced by the sigmoidal force-response functions observed in squirrel monkey primary afferent recordings (Fernandez and Goldberg, 1976b); as such, ε may be sent into the cerebellum directly.

### Limitations and future work

There are several limitations in our current study that naturally lead to future experiments. One limitation is that our assumption of innate otolith asymmetries stems from a perceptual measure of ocular positioning misalignments. Since we cannot explicitly record vestibular afferent activity in humans, we could instead perform a series of tests, including VAN and TAN, to substantiate our otolith asymmetry hypothesis. For example, recording the eye movements directly would provide a measure of the pure motor output in response to altered gravity. Incorporating cervical and ocular vestibular-evoked myogenic potentials may enable us to pair utricular- vs. saccular-driven oculomotor control. Furthermore, additional non-1 g VAN and TAN data are needed to verify our model. With only our two non-1 g data points, there are multiple possible parameter values. Hence this work would benefit from future parabolic flight experiments that test, for example, in Lunar and Martian g-levels. Thirdly, previous investigators have found an interesting relationship between the magnitude of ocular misalignments in novel g-levels and motion sickness (both parabolic flight motion sickness and SMS) (Kornilova et al., 1983; Vogel and Kass, 1986; Lackner et al., 1987; Diamond et al., 1990; Diamond and Markham, 1991, 1992b). Since none of our non-astronaut subjects experienced adverse symptoms inflight, we could not compare their ocular misalignments to motion sickness susceptibility. Future experiments that incorporate either astronauts or non-astronauts who are more susceptible to terrestrial motion sickness might facilitate similar correlations to be made.

Finally, one important aspect not detailed in our model, nor in the von Baumgarten and Thümler one, is the time-dependent nature of these g-level dependent compensations. We know from spaceflight literature that astronauts adapt sensorimotor responses over various timescales to optimize performance in the novel g-levels (Michel et al., 1976; von Baumgarten, 1986; Baroni et al., 2001; Williams et al., 2009). Furthermore, parabolic flight research has demonstrated that repeated exposure to alternating g-levels also leads to adaptive responses over time (Graybiel and Lackner, 1983; Oman et al., 1996; Karmali, 2007). However, because no systematic adaptive responses were captured in our VAN and TAN parabolic flight data, it was not possible to incorporate timing information into our model. Future experiments that capture ocular misalignments over longer periods of time (e.g., across multiple, consecutive parabolic flights) would enable this incorporation.

### Conflict of interest statement

The authors declare that the research was conducted in the absence of any commercial or financial relationships that could be construed as a potential conflict of interest.

## Footnotes

^1^Since our experiment did not include astronaut test subjects, we could not correlate our results with SMS susceptibility, as has been done by previous investigators. Our intent was simply to verify that our device was sensitive to detect the ocular misalignments observed by others in novel gravity environments.

^2^Note that a simple multiplicative model (e.g., gain change) would not enable adequate compensation in 0 g, when no signals are from either side.

^3^In healthy individuals, it is presumed that the magnitude of this asymmetry is relatively small, and hence it is likely that *k* is very close to 1. However, the model allows *k* to vary between 0 and 1, and as such, it can accommodate individuals with unusually large asymmetries, including those with complete unilateral vestibular loss.

^4^While *r_L_* and *r_R_* are likely to be similar in magnitude, the model allows for unequal magnitudes, as slight differences may be present due to innate asymmetries between the left and right sides.
